# Cognitive impairment, depression, and fatigue in post-COVID and post-vaccination syndrome: a large-scale cross-sectional study

**DOI:** 10.1007/s00406-025-02144-3

**Published:** 2025-11-19

**Authors:** Evelyne Hanc, Katharina Koller, Regina Herold, Yesim Erim, Eva Morawa

**Affiliations:** 1 Department of Psychosomatic Medicine and Psychotherapy, University Hospital of Erlangen, Friedrich-Alexander University Erlangen-Nürnberg (FAU), Schwabachanlage 6, 91054 Erlangen, Germany; 2 Post-COVID Center of the University Hospital of Erlangen, Erlangen, Germany

**Keywords:** Post-COVID syndrome, Post-Vaccination syndrome, Cognitive impairments, Fatigue, Depression, Neuropsychological assessment

## Abstract

**Background:**

Persistent cognitive difficulties are among the most prevalent symptoms observed after COVID-19. This study examined the neuropsychological characteristics, mental health and risk factors associated with cognitive impairment in patients with Post-Acute Sequelae of COVID-19 (PASC). In addition, differences regarding the cognitive dysfunctions between individuals with PASC and Post-Vaccination Syndrome (PVS) were explored.

**Methods:**

Participants were consecutively recruited at the Post-COVID Center of the University Hospital Erlangen in Germany (12/2022–02/2025). Assessments included a broad neuropsychological assessment: d2 Test of Attention, digit span backwards from the Wechsler Memory Scale-Revised (WMS-R) and module 1 (formal lexical fluency) of the Regensburger Verbal Fluency Test (RWT), along with assessments of depression, fatigue, and inflammatory markers (C-reactive protein).

**Results:**

The sample (N = 793, mean age: 46.1 ± 12.4 years; 66.8% women) included n = 723 PASC patients and n = 70 PVS patients. In the total sample, deficits were most frequent in working speed (61.7%), attention (54.7%) and verbal fluency (48.8%); 30.3% showed multidomain impairment. Clinically significant depressive symptoms (64.5%) and fatigue (92.4%) were common. Cognitive patterns, depressive symptoms and fatigue in the PVS group closely resembled those observed in PASC. In PASC, lower education, older age, depressive symptoms, and elevated CRP were associated with cognitive impairment.

**Conclusion:**

Cognitive impairment is highly prevalent in PASC and PVS and appears to be influenced by both psychological and biological factors. Similar patterns identified in PVS suggest possible shared mechanisms. Further research is needed to clarify trajectories and optimize treatment strategies.

**Supplementary Information:**

The online version contains supplementary material available at 10.1007/s00406-025-02144-3.

## Introduction

COVID-19, the most recent global pandemic, has had far-reaching effects that extend beyond the acute phase of infection. The World Health Organization (WHO) defines post-acute sequelae of COVID-19 (PASC) as the persistence or emergence of new symptoms at least three months after the initial SARS-CoV-2 infection, lasting for a minimum of two months and not attributable to other causes [1, 2].

PASC presents with a broad and heterogeneous range of symptoms affecting multiple organ systems, making diagnosis particularly challenging. More than 200 persistent symptoms have already been identified [3], with fatigue, dyspnea, and cognitive impairment being among the most common [4–7]. These symptoms may be variable or persistent, newly emerging, or continuing as long-term complications from the acute phase, with varying severity [8].

A significant proportion of individuals continue to experience cognitive complaints long after the acute infection. Numerous studies have found that approximately 20% of patients exhibit objectively measurable impairments in cognitive performance, making it the second most frequent symptom of PASC after fatigue [7, 9]. Nearly half of these patients are unable to return to work, with this proportion increasing to 90% among intensive care unit (ICU) survivors [10]. In addition to cognitive impairments, many PASC patients experience mental health comorbidities, such as sleep disturbances, depressive symptoms, and anxiety [5, 11]. These psychological and cognitive sequelae often coexist, creating a complex clinical picture that complicates recovery and treatment. As a result, persistent cognitive deficits pose significant long-term challenges, substantially impairing daily functioning, occupational performance, and overall quality of life [5, 12, 13].

The extent and pattern of cognitive dysfunction following a SARS-CoV-2 infection are highly heterogeneous [14]. Emerging evidence suggests that these impairments often manifest as deficits in memory, attention, processing speed, and executive functioning [11, 15–18]. Various meta-analyses have identified the most prevalent neurocognitive symptoms in PASC patients, including "brain fog," memory problems, and attention deficits. Impairments in executive functions and processing speed are also frequently observed, whereas visuospatial dysfunctions appear to be relatively rare [16, 17, 19, 20].

The trajectory of cognitive impairments in PASC remains inconsistent across studies, likely due to methodological variability and heterogeneous sample compositions. While some studies report a significant decrease in cognitive deficits over time [21, 22], others find an increase [23] or observe an initial rise (3 and 6 months after acute infection) followed by a later decline (9 and 12 months later) [22]. Given these variations, identifying both psychosocial and biological risk factors for persistent cognitive deficits is crucial.

In addition to psychosocial and clinical variables, biological markers have increasingly come into focus as potential indicators of ongoing inflammation and neurocognitive dysfunction in PASC. Elevated levels of C-reactive protein (CRP) have been observed in individuals with PASC symptoms and may contribute to persistent cognitive and psychological impairments [24, 25].

Risk factors for PASC include older age, female gender, smoking, higher BMI, pre-existing medical conditions, and hospitalization during the acute COVID-19 phase [26]. In addition, male gender, older age, obesity, mental health symptoms, and lower educational attainment (< 12 years) at time of assessment specifically increase the risk for persistent cognitive impairment [27–29]. Encouragingly, vaccination has been shown to significantly decrease the risk of developing PASC, including cognitive symptoms [23, 30, 31], while recurrent SARS-CoV-2 infections do not appear to substantially impact cognitive recovery outcomes [28].

Despite the growing recognition of these neurocognitive and psychological impairments, there is currently no established causal treatment for PASC. Identifying the nature and extent of cognitive impairments, as well as potential risk factors, is essential for developing targeted diagnostic and therapeutic approaches. To address these gaps, this cross-sectional study aimed to provide a detailed characterization of cognitive impairment in PASC patients. Using a comprehensive battery of validated neuropsychological tests, we assessed a cohort of patients with persistent PASC symptoms. The study also used regression analyses to identify potential risk factors for cognitive impairment. With a primary focus on PASC, this study also examines a subset of individuals with Post-Vaccination syndrome (PVS) to assess potential neurocognitive similarities with PASC. Symptoms emerging after a COVID-19 vaccination can resemble those of PASC [32], and it is assumed that similar pathophysiological mechanisms may be involved. Given the limited research on this condition, these findings offer important initial insights into its cognitive characteristics.

This study builds upon the sample previously analyzed by Morawa et al. (2023) [33] with an expanded cohort. It employs a more time-efficient neuropsychological test battery and considers additional factors that may be associated with cognitive impairment. Notably, this study benefits from a particularly large sample size and, for the first time, directly compares cognitive performance between individuals with PASC and those with PVS.

Specifically, the study aims to examine:

1. The type and frequency of cognitive impairment;
2. Gender-specific differences in cognitive impairment;
3. Risk factors associated with cognitive impairment, including psychosocial (gender, education), clinical (time since acute infection) and biological factors (elevated CRP levels);
4. Group differences between PASC and PVS.

We hypothesized that impairments would most frequently occur in the domains of word fluency, attention and processing speed. Additionally, we expected a significant proportion of patients to exhibit clinically relevant levels of depressive symptoms and fatigue. Furthermore, we anticipated that cognitive impairment in PASC patients would be associated with gender, older age, lower educational level, higher BMI, smoking, longer duration of PASC symptoms, depressive symptoms and fatigue. As an additional objective, we postulated that elevated inflammatory markers (CRP) would be associated with cognitive performance. Given the exploratory nature of the last research question, no specific hypotheses were formulated regarding group differences between PASC and PVS.

## Methods

### Statement of ethics

The present study was approved by the Ethics Committee of the Medical Faculty of the Friedrich-Alexander University Erlangen-Nürnberg (FAU) (reference number: 22-443-B). All respondents provided their written informed consent prior to participation.

### Data collection and participants

Participants were included consecutively between 12/2022 and 02/2025 at the Post-COVID Center of the University Hospital of Erlangen, a multidisciplinary outpatient clinic specializing in patients with PASC. They underwent a comprehensive neuropsychological assessment by trained psychologists, as well as a medical and psychosomatic examination by various specialists (rheumatology, immunology, ophthalmology). Referral to the Post-COVID Center was possible only through a general practitioner and required a confirmed SARS-CoV-2 infection and persistent symptoms lasting at least three months. Patients with PVS were included if they reported new-onset symptoms in a clear temporal association with a COVID-19 vaccination, as documented in the medical history at the Post-COVID Center. Diagnostics included laboratory analyses, an electrocardiography, cardiac ultrasound and pulmonary function testing. Participants also completed an online-questionnaire assessing sociodemographic, health-related, and psychological variables (e.g. depression, anxiety, social support). Inclusion criteria were PASC or PVS symptoms, a minimum age of 18 years and sufficient proficiency in the German language. Individuals with severe neurological or psychiatric disorders (e.g. dementia) were excluded from participation. Further methodological details are reported in Morawa et al. (2023) [33].

### Measures

#### Sociodemographic variables

Data were collected on *gender*, *age*, *marital status,* *employment*, and *parental status*. *Educational level* was categorized into five groups based on the structure of the International Standard Classification of Education (ISCED) [34]: (1) No formal education = no school or vocational qualification; (2) Low education = lower secondary school without vocational training; (3) Medium education = general school certificate with vocational qualification (e.g. apprenticeship, master craft training, advanced vocational school); (4) High education = higher secondary certificate (e.g. Abitur) with vocational training; (5) Tertiary education = academic degree from a university or university of applied sciences.

#### Health-related variables

To identify potential risky health-related behaviors, *smoking status* was assessed via self-report and categorized as never smoker, current smoker, occasional smoker, or former smoker. In addition, *Body Mass Index* (BMI) was calculated using self-reported height and weight (kg/m^2^).

Based on the WHO classification [35], participants were categorized as underweight (< 18.5), normal weight (18.5–24.9), pre-obese (25.0–29.9), or obese, with obesity further classified into class I (30.0–34.9), class II (35.0–39.9), and class III (≥ 40.0).

#### Biological markers

In a subset of the sample, *inflammatory markers* were assessed as potential indicators of systemic immune activation. C-reactive protein (CRP) was analyzed from routine blood samples. Based on clinical reference ranges, CRP levels > 5.2 mg/l were classified as elevated.

#### COVID-19-related variables

The date of the SARS-CoV-2 infection was extracted from medical records. Based on this information, the *time since the acute infection* could be determined. Additionally, *course of the acute SARS-CoV-2 infection* was categorized into four types: 1. asymptomatic; 2. symptomatic, therapy at home or outpatient; 3. symptomatic, inpatient therapy without intensive care admission; and 4. symptomatic, inpatient therapy with intensive care admission. In addition, the *vaccination status* (vaccinated vs. not vaccinated) was also transferred from medical records. In the present study, “vaccinated” referred to having received at least one COVID-19 vaccination, independent of the total number of doses administered.

#### Cognitive assessments

The neuropsychological assessment consisted of the following validated tests: the digit span backwards from the Wechsler Memory Scale-Revised for assessing the working memory (WMS-R) [36], the d2 Test of Attention for working speed and attention [37] and module 1 of the Regensburger Verbal Fluency Test (RWT) [38], which examines the formal lexical (= phonemic) fluency. Patients were asked to name as many words with the initial letter “k” as possible within two minutes. We defined scores below the cut-offs as cognitive impairment as described in the Data Analysis section.

#### Depressive symptoms

The Patient Health Questionnaire-9 (PHQ-9) was used to measure depression symptoms. The range of sum scores is 0–27. To identify likely cases of clinically significant levels of depression symptoms, a cut-off value of ≥ 10 was proposed [39]. The validated German version of the PHQ-9 in this study demonstrated good internal consistency (Cronbach's Alpha = 0.80).

#### Fatigue

The Fatigue Severity Scale (FSS) was used to assess the severity of fatigue. The scale consists of 9 items, each rated on a 7-point Likert scale. A cut-off score of ≥ 36 is commonly used to indicate clinically significant fatigue [40]. The German version of the FSS used in this study showed very good internal consistency, with a Cronbach's Alpha of 0.92.

### Data analysis

Cognitive impairment in the neuropsychological tests was determined by comparing individual scores with normative data from the respective test manuals. For the d2 Test, raw scores were transformed into age-adjusted standard values, with scores < 95 classified as below average. For the RWT and the digit span backwards (WMS-R), raw scores were age-adjusted and converted into percentiles or percentage ranks (PR), with the 16th percentile (WMS-R) or PR (RWT) serving as the cut-off point for impairment.

Missing data were imputed if less than 20% of items on a questionnaire were missing, which was only the case for one participant on the FSS. Descriptive statistics (absolute and relative frequencies) were calculated to describe sociodemographic and COVID-related characteristics and the proportion of the sample with cognitive impairment. Comparisons with healthy controls and between the PASC and PVS group were conducted using one-sample t-tests or t-tests for independent samples, respectively. Gender differences in cognitive performance were examined using chi-square tests. Separate analyses were conducted for each cognitive test to compare the proportion of male and female participants performing below average. Associations between inflammatory markers and cognitive performance were examined using chi-square tests. Binomial tests were conducted to compare the proportions of BMI categories and smoking behavior in the study sample with corresponding population values. Binary logistic regression analyses were used to identify factors associated with cognitive impairment in the assessed cognitive domains. Odds ratios (OR) and their respective confidence intervals (CI), as well as effect sizes were reported with Cohen's d (small: d ≥ 0.2, medium: d ≥ 0.5, large: d ≥ 0.8)or Cramér's V (small: V ≥ 0.1, medium: V ≥ 0.3, large: V ≥ 0.5). The level of significance was set to p < 0.05 (two-sided). Valid percentages are reported. Data were analyzed using SPSS V.28 (IBM Corporation, Armonk, New York).

## Results

A total of N = 810 patients were consecutively recruited. Three patients were excluded due to a post-viral syndrome unrelated to COVID-19 and six patients because of symptoms of unclear origin that could not be clearly attributed to either PASC or PVS. Three patients were excluded due to neurological conditions, and five for insufficient German language proficiency. This resulted in a final sample of N = 793 patients, including 723 patients with PASC (91.2%) and 70 with PVS (8.8%). For the d2 test of attention, 12 patients were excluded due to missing visual aids and one due to acute visual impairment. For the digit span backwards task, 11 patients were excluded because of self-reported dyscalculia. Final sample sizes for each neurocognitive assessment are reported in Table 3.

An additional 20 patients who had presented at the Post-COVID Center but did not complete either the questionnaires or the neurocognitive assessment were classified as nonresponders and not included in the sample. Nonresponders (n = 20) did not differ significantly from included participants (n = 793) in age (*t*(811) = -1.95, *p* = 0.051; d = 0.44) or gender (χ^2^(1) = 2.48, *p* = 0.115; Cramér’s V = 0.06).

### Sociodemographic variables

The sociodemographic characteristics of the study sample are presented in Table 1. The total sample consisted predominantly of women (66.8%, n = 530), with a mean age of 46.1 years (SD = 12.4). Educational attainment was generally high, with 32.7% (n = 256) holding a university degree and 43.4% (n = 344) reporting a medium-level vocational qualification. Regarding employment status, 25.9% (n = 205) were employed full-time, while 10.6% (n = 84) were on sick leave or unable to work at the time of assessment. Sociodemographic characteristics were comparable between the PASC and PVS groups.

**Table 1:** Socio-demographic characteristics of the study sample

Variables	Total sample (N = 793)	PASC patients (n = 723)	Post-Vacc patients (n = 70)
Gender, n (%)			
Women	530 (66.8)	491 (67.9)	39 (55.7)
Men	263 (33.2)	232 (32.1)	31 (44.3)
Age, years			
M (SD)	46.1 (12.4)	46.0 (12.2)	47.0 (14.1)
Range	18-82	18-79	18-82
Marital status, n (%)			
Single without partnership	116 (14.6)	105 (14.5)	11 (15.7)
Single with partnership	134 (16.9)	123 (17.0)	11 (15.7)
Married	401 (50.8)	368 (50.9)	33 (47.1)
Divorced/ in separation	73 (9.3)	67 (9.2)	6 (8.6)
Missing	69 (8.7)	60 (8.3)	9 (12.9)
Children, n (%)			
Yes	443 (55.9)	407 (56.3)	36 (51.4)
No	281 (35.4)	256 (35.4)	25 (35.7)
Missing	69 (8.7)	60 (8.3)	9 (12.9)
Educational level^a^, n (%)			
No formal education	3 (0.4)	1 (0.1)	2(2.9)
Low education	12 (1.5)	10 (1.4)	2 (2.9)
Medium education	344 (43.4)	320 (44.3)	24 (34.3)
High education	106 (13.4)	98 (13.6)	8 (11.4)
Tertiary education	256 (32.7)	234 (32.4)	25 (35.7)
Missing	69 (8.7)	60 (8.3)	9 (12.9)
Employment status, n (%)			
Full-time employed	205 (25.9)	188 (26.0)	17 (24.3)
Part-time employed	190 (24.0)	179 (24.8)	11 (15.7)
Sick on leave/ unable to work	84 (10.6)	78 (10.8)	6 (8.6)
Unemployed	96 (12.2)	87 (12.0)	9 (12.9)
Retired/ pensioned	114 (14.4)	102 (14.1)	12 (17.1)
In education/ training	10 (1.3)	8 (1.1)	2 (2.9)
Other	25 (3.2)	21 (2.9)	4 (5.7)
Missing	69 (8.7)	60 (8.3)	9 (12.9)

^a^Educational levels according to the International Standard Classification of Education (ISCED): No formal education = no school or vocational qualification; Low education = lower secondary school without vocational training; Medium education = general school certificate with vocational qualification (e.g. apprenticeship, master craft training, advanced vocational school); High education = higher secondary certificate (e.g. Abitur) with vocational training; Tertiary education = academic degree from a university or university of applied sciences

### Health related-variables

Mean BMI in the total sample was 26.5 kg/m^2^ (SD = 5.8; see Table 2). Most participants were classified as either normal weight (38.6%) or pre-obese (27.9%), totaling 66.5%. An additional 21.4% were classified as obese (class I–III). Compared to population-based reference data [41] (mean BMI = 26.0 kg/m^2^), the sample showed a slightly higher average BMI, t(721) = 2.45, *p* = 0.015, with a small effect size (Cohen’s d = 0.09). The distribution of BMI categories also differed significantly from the expected population proportions, χ^2^(3) = 34.32, *p* < 0.001, with a small effect (Cramér’s V = 0.13). The sample included more participants classified as obese and fewer in the normal and pre-obese ranges than expected.

**Table 2:** Health and COVID-related characteristics of the study sample

Variables	Total sample (N = 793)	PASC patients (n = 723)	PVS patients (n = 70)
BMI, n (%)			
M (SD)	26.5 (5.8)	26.5 (5.8)	26.3 (5.5)
Underweight (< 18.5)	25 (3.2)	23 (3.2)	2 (2.9)
Normal weight (18.5–24.9)	306 (38.6)	282 (39.0)	24 (34.4)
Pre-obese (25.0–29.9)	221 (27.9)	200 (27.7)	21 (30.0)
Obese class I (30.0–34.9)	109 (13.7)	100 (13.8)	9 (12.9)
Obese class II (35.0–39.9)	45 (5.7)	41 (5.7)	4 (5.7)
Obese class III (≥ 40.0)	12 (2.0)	12 (2.1)	1 (1.4)
Missing	71 (9.0)	62 (8.6)	9 (12.9)
Smoking habits, n (%)			
Never smoker	481 (60.7)	441 (61.0)	40 (57.1)
Current smoker	45 (5.7)	40 (5.5)	5 (7.1)
Occasional smoker	14 (1.8)	14 (1.9)	0 (0)
Former smoker	168 (21.2)	156 (21.6)	12 (7.1)
Missing	85 (10.7)	72 (10.0)	13 (18.6)
Vaccination status, n (%)			
Yes, vaccinated	705 (88.9)	635 (87.8)	70 (100)
No vaccination	44 (5.5)	44 (6.1)	0 (0)
Missing	44 (5.5)	44 (6.1)	0 (0)
Time since the SARS-CoV-2 infection (months)	n = 766	n = 702	n = 64
M (SD)	24.5 (11.38)	24.2 (11.50)	27.5 (9.66)
Range	3 – 58	3 – 58	10 – 57
Course of the acute SARS-CoV-2-infection, n (%)			
Asymptomatic	25 (3.2)	16 (2.2)	9 (12.9)
Symptomatic, therapy at home or outpatient	623 (78.6)	579 (80.1)	44 (62.9)
Symptomatic, inpatient therapy without intensive care admission	45 (5.7)	43 (5.9)	2 (2.9)
Symptomatic, inpatient therapy with intensive care admission	12 (1.6)	12 (1.6)	0 (0)
Missing	88 (11.1)	73 (10.1)	15 (21.4)

Regarding smoking behavior, most participants had never smoked (60.7%) or were former smokers (21.2%), while only a minority reported current (5.7%) or occasional smoking (1.8%). Compared to national data from the Epidemiological Survey of Substance Abuse 2021 [42] (22.7% active smokers), the proportion of current or occasional smokers in the sample was significantly lower (7.5%), χ^2^(1) = 83.39, *p* < 0.001, with a moderate effect size (Cramér’s V = 0.34), indicating substantially reduced tobacco use in the present study cohort.

### COVID-19-related variables

The average time since the SARS-CoV-2 infection prior to study participation was 24.5 months (SD = 11.4; see Table 2). A large proportion of patients reported a symptomatic course of infection, with the majority receiving home or outpatient treatment. Regarding vaccination status, 88.9% (n = 705) of the total sample had received at least one COVID-19 vaccination.

### Biological parameters

Data on biological parameters were available in 467 patients CRP. Elevated CRP levels (> 5.2 mg/l) were found in 99 patients (21.2%, M = 12.8, SD = 15.4; range 0.2–92.6 mg/l). In the PASC group, 21.5% of patients showed elevated CRP levels (M = 6.17, SD = 7.35; range 0.2–92.6 mg/l), while in the PVS group, 16.7% had elevated values (M = 5.56, SD = 1.62; range 5.1–15.4 mg/l).

### Depression

Analyses were based on a total of 732 participants. The prevalence of probable depression (cut-off ≥ 10) was 64.5% (n = 472). The mean score for depressive symptoms was M = 11.5 (SD = 5.1). Mean PHQ-9 scores were 11.6 (SD = 5.1) in the PASC group and 10.4 (SD = 5.0) in the PVS group. An independent samples t-test showed no significant difference between groups, t(730) = –1.81, *p* = 0.070, d = 0.24.

### Fatigue

Analyses included 719 participants. A total of 92.4% of participants (n = 664) reported clinically relevant levels of fatigue (cut-off ≥ 36). The average symptom severity was M = 53.9 (SD = 10.5). The mean FSS score was 53.8 (SD = 10.6) in the PASC group and 54.3 (SD = 9.0) in the PVS group. The difference between groups was not statistically significant, t(717) = 0.36, *p* = 0.722, d = 0.05.

### Cognitive assessments

In Table 3, the results of the neuropsychological assessment are presented (as percentages of age-adjusted test scores below the norm and mean scores and standard deviations of the tests).

**Table 3:** Neurocognitive test performance in PASC and PVS patients

Cognitive tests and functions		Total sample			PASC patients			PVS patients		Comparison PASC vs. PVS
	n	Distribution of age-adjusted test scores below the norm, n (%)	M (SD)	n	Distribution of age-adjusted test scores below the norm, n (%)	M (SD)	n	Distribution of age-adjusted test scores below the norm, n (%)	M (SD)	p-value for independent samples t-test (effect size)
WMS-R										
Digit span backwards (short term and working memory)	775	272 (35.1)	6.4 ± (2.0)	709	253 (35.7)	6.3 ± (2.0)	66	19 (28.8)	6.6 ± (2.1)	0.263 (0.14)
d2										
Attention	746	408 (54.7)	125.0* (41.8)	680	369 (54.3)	125.5* (41.7)	66	39 (59.1)	119.1* (43.2)	0.238 (0.15)
Working speed	745	460 (61.7)	142.1 + (42.9)	679	415 (61.1)	142.2 + (42.5)	66	45 (68.2)	141.5 + (47.9)	0.905 (0.02)
RWT										
Formal lexical fluency	695	339 (48.8)	17.5# (6.9)	632	308 (48.7)	17.5# (6.2)	63	31 (49.2)	17.2# (6.3)	0.720 (0.05)

*WMS-R* Wechsler Memory Scale-Revised, *d2* d2 Test of Attention, *RWT* Regensburger Verbal Fluency Test; valid percentages are presented; ± number of correctly named digit spans backwards

*Number of correctly marked d2-symbols minus the number of incorrectly marked symbols (KL)

+Number of processed target objects (BZO)

^#^Number of unique and correctly named words with the initial letter “k”

An impairment in at least one of the four assessed cognitive domains was observed in 79.7% (n = 632) of the patients. Additionally, 30.3% (n = 240) of patients exhibited multi-domain impairment (≥ 3 tests below average).

In the d2 test of attention, 54.7% (n = 408) of the total sample scored below the norm (SW < 95) in attention, and 61.7% (n = 460) in working speed. Among PASC patients, 54.3% (n = 369) showed reduced attention and 61.1% (n = 415) reduced working speed; in PVS patients, 59.1% (n = 39) and 68.2% (n = 45), respectively. Regarding verbal fluency (RWT), 48.8% (n = 339) of the total sample scored below the norm in the formal lexical subtest (< 16 PR). The prevalence of deficits was 48.7% (n = 308) in PASC patients and 49.2% (n = 31) in PVS patients. In the digit span backwards subtest from the WMS-R, indicating short-term and working memory capacity, 35.1% (n = 272) of the total sample performed below average (< 16 percentil). This was observed in 35.7% (n = 253) of PASC patients and in 28.8% (n = 19) of PVS patients. No statistically significant differences were found between the PASC and PVS groups in any of the assessed cognitive domains (effect sizes d = 0.02–0.15).

### Gender-specific analyses

**Fig. 1:**
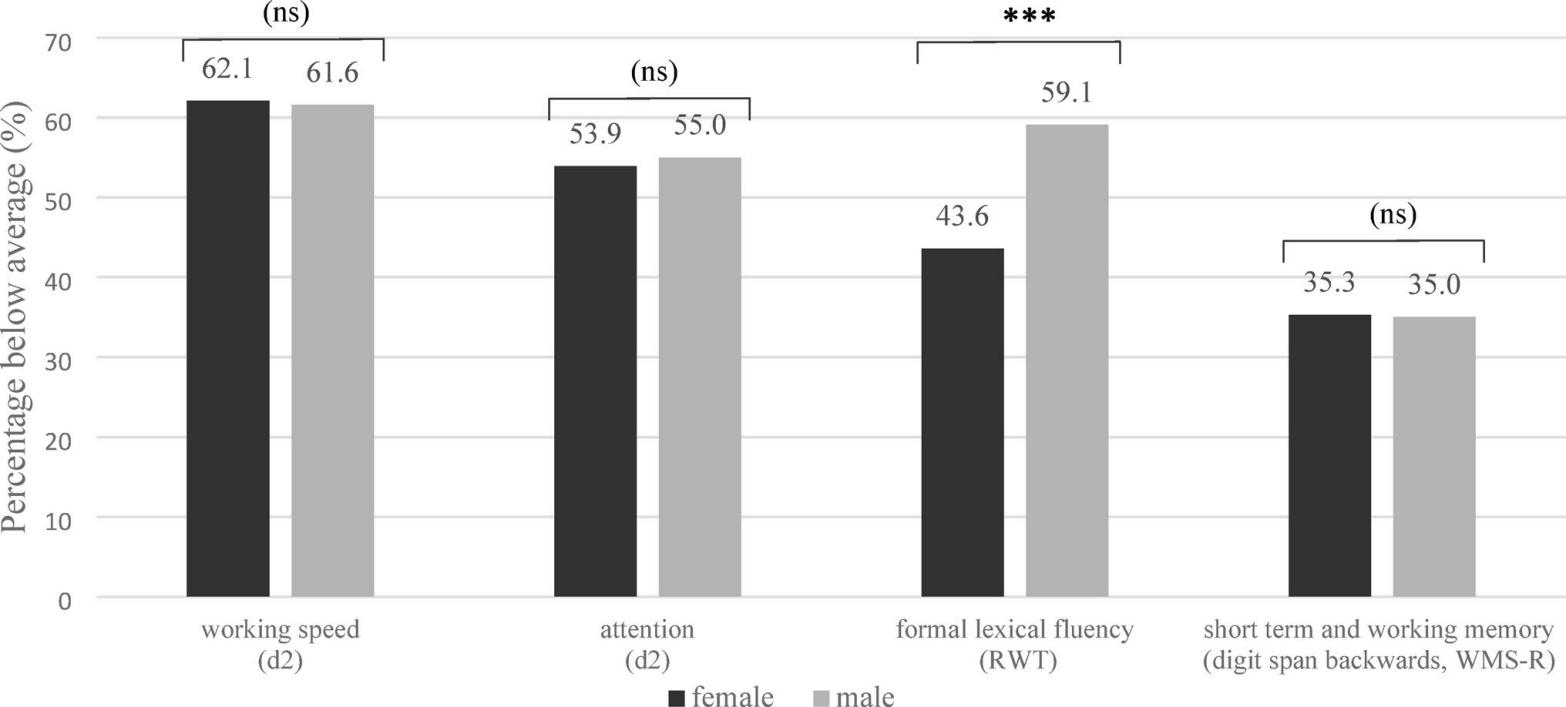
**Percentage of participants with below-average test performance by gender (total sample)**. *d2* d2 Test of Attention, *RWT* Regensburger Verbal Fluency Test, *WMS-R* Wechsler Memory Scale-Revised; valid percentages are presented. *** *p* < 0.001, (ns) = not significant

Figure 1 shows the percentage of male and female participants in the total sample who performed below average in each cognitive domain. Chi-square analyses revealed a statistically significant gender difference in formal lexical verbal fluency (RWT), with a higher proportion of men performing below average compared to women, χ^2^(1) = 14.72, *p* < 0.001, Cramér’s V = 0.15. No significant differences were found in attention (d2 test: *p* = 0.783) and working speed (d2 test: *p* = 0.877), and in working memory (digit span backwards, WMS-R: *p* = 0.943). Subgroup analyses for PASC and PVS patients showed a gender difference in formal lexical verbal fluency only among PASC patients (*p* < 0.001; Cramér’s V = 0.15), while no significant gender differences were observed in any other cognitive domain or in the PVS group (data not shown).

### Comparisons with healthy controls

When comparing the cognitive performance of the study sample with normative data from healthy controls (see Table 4), significantly lower scores were found in the cognitive domains of attention, working speed and formal lexical fluency. Both age groups (20–39 and 40–60 years) in the study sample showed significantly reduced performance in attention and working speed (d2 test) compared to healthy controls, with particularly large effect sizes in the older age group. Formal lexical fluency (RWT) was also significantly impaired in the study sample when compared to controls. Subgroup analyses revealed consistent cognitive impairments among PASC patients across all comparisons. In contrast, significant differences in cognitive performance among PVS patients were predominantly observed in the older age group, while the younger group showed no significant differences from healthy controls in attention and working speed. Notably, no statistically significant differences were found between the PASC and PVS groups in any of the assessed cognitive domains.

**Table 4:** Comparison of cognitive performance between study sample and healthy controls, total sample and subsamples (PASC vs. PVS)

Cognitive functions (neuropsychological tests)	Healthy controls: Age group/ mean age (SD) [N]	Total study sample: Age group/ mean age (SD) [N]	Healthy controls: Mean score (SD)	Total study sample: Mean score (SD)	Comparison p-value (effect size)
Attention (d2)^a^	20–39 yrs. [708]	20–39 yrs. [227]	158.6* (29.4)	144.2* (41.0)	** < 0.001 (0.35)**
Attention (d2)^a^	40–60 yrs. [268]	40–60 yrs. [424]	148.1* (27.4)	116.6 + (39.9)	** < 0.001 (0.79)**
Working speed (d2)^a^	20–39 yrs. [708]	20–39 yrs. [227]	175.7 + (29.6)	160.0* (43.0)	** < 0.001 (0.37)**
Working speed (d2)^a^	40–60 yrs. [268]	40–60 yrs. [423]	167.7 + (29.2)	133.1 + (40.4)	** < 0.001 (0.86)**
Formal lexical fluency (words with initial letter “k”) (RWT)^b^	40.1 (12.5) [17]	46.3 (12.6) [695]	22.3# (9.3)	17.5# (6.2)	** < 0.001 (0.78)**

Cognitive functions (neuropsychological tests)	Study sample: Age group/ mean age (SD) [n (PASC)/n (PVS)]	Study sample (PASC): Mean Score (SD)	Comparison p-value PASC vs. healthy controls (effect size)	Study sample (PVS): Mean Score (SD)	Comparison p-value PVS vs. healthy controls (effect size)	Comparison p-value PASC vs. PVS (effect size)
Attention (d2)^a^	20–39 yrs. [205/22]	144.3 (40.7)	** < 0.001 (0.35)**	143.1 (43.6)	0.055 (0.36)	0.893 (0.03)
Attention (d2)^a^	40–60 yrs. [391/33]	117.5 (40.0)	** < 0.001 (0.77)**	106.2 (38.0)	** < 0.001 (1.10)**	0.116 (0.29)
Working speed (d2)^a^	20–39 yrs. [205/22]	159.6 (42.4)	** < 0.001 (0.38)**	162.8 (48.5)	0.114 (0.27)	0.742 (0.07)
Working speed (d2)^a^	40–60 yrs. [390/33]	134.1 (40.5)	** < 0.001 (0.83)**	121.0 (38.1)	** < 0.001 (1.23)**	0.075 (0.32)
Formal lexical fluency (words with initial letter “k”) (RWT)^b^	PASC 46.3 (12.4) [632] PVS 46.2 (14.4) [63]	17.5 (6.2)	** < 0.001 (0.78)**	17.2 (6.3)	** < 0.001 (0.81)**	0.720 (0.05)

*d2* d2 Test of Attention, *RWT* Regensburger Verbal Fluency Test

*Number of correctly marked d2-symbols minus the number of incorrectly marked symbols (KL)

+Number of processed target objects (BZO)

^#^Number of unique and correctly named words with the initial letter “k”

^a^Reference for healthy controls: Brickenkamp R, Schmidt-Atzert L, Liepmann D. Test d2 – Revision. Aufmerksamkeits- und Konzentrationstest. Göttingen u.a.: Hogrefe; 2010

^b^Reference for healthy controls: Aschenbrenner S, Tucha O, Lange KW. Regensburger Wortflüssigkeits-Test (RWT). Göttingen u.a.: Hogrefe; 2001. After Bonferroni-Holm correction, all comparisons (n = 15) between PASC and controls remained significant (< 0.001); in the PVS group, three of five comparisons remained significant, while PASC vs. PVS comparisons remained non-significant

### Association between inflammatory markers and cognitive performance

Chi-square analyses of the total sample revealed that elevated CRP levels were significantly associated with below-average performance in attention (d2 test; n = 434; χ^2^(1) = 4.47, *p* = 0.034, φ = 0.101), working memory (digit span backwards, WMS-R; n = 457; χ^2^(1) = 5.40, *p* = 0.020, φ = 0.109) and multidomain impairment (n = 467; χ^2^(1) = 5.93, *p* = 0.015, φ = 0.113), all indicating small effects. A comparison between PASC and PVS patients showed no significant group difference in the frequency of elevated CRP levels (χ^2^(1) = 0.39, *p* = 0.530, φ = 0.029).

### Predictors of cognitive impairment

Binary logistic regression analyses were conducted for each cognitive domain and for multidomain impairment with cognitive performance as the criterion variable (see Table 5). The same set of predictors was entered into all models: age, gender, education level (dichotomized), time since acute infection, BMI (dichotomized), smoking status (dichotomized), depressive symptoms (dichotomized), and fatigue (dichotomized).

**Table 5:** Binary logistic regression analyses in the assessed cognitive domains (respective age-adjusted scores below the test-specific norms as criterion variable) in PASC patients

Predictors	Model 1: Attention (d2) (n = 586)		Model 2: Working speed (d2) (n = 586)		Model 3: Formal lexical fluency (RWT) (n = 545)		Model 4: Short-term/ working memory (WMS-R) (n = 616)		Model 5: Multi-domain cognitive dysfunction (≥ 3 tests below average) (n = 628)	
	OR [CI]	p	OR [CI]	p	OR [CI]	p	OR [CI]	p	OR [CI]	p
Age	1.02 [1.00–1.03]	**0.036**	1.02 [1.01–1.04]	**0.007**	0.96 [0.94–0.97]	** < 0.001**	1.01 [1.00–1.03]	0.080	1.00 [0.98–1.02]	0.938
Gender*	0.94 [0.63–1.39]	0.745	1.12 [0.75–1.67]	0.583	2.01 [1.35–3.00]	** < 0.001**	0.98 [0.98–1.43]	0.899	1.29 [0.88–1.90]	0.193
Education^#^	0.37 [0.26–0.52]	** < 0.001**	0.38 [0.26–0.55]	** < 0.001**	0.57 [0.39–0.82]	**0.002**	0.55 [0.39–0.78]	** < 0.001**	0.52 [0.36–0.75]	** < 0.001**
Time since acute SARS-CoV-2-infection	1.02 [1.01–1.04]	**0.004**	1.01 [1.00–1.03]	0.124	1.01 [0.99–1.02]	0.490	1.02 [1.00–1.04]	**0.017**	1.03 [1.01–1.04]	** < 0.001**
BMI +	1.07 [0.70–1.64]	0.760	1.14 [0.73–1.78]	0.565	1.72 [1.13–2.63]	**0.012**	1.71 [1.15–2.55]	**0.008**	1.31 [0.86–1.98]	0.206
Smoking habits^§^	1.08 [0.74–1.58]	0.695	1.03 [0.70–1.53]	0.866	0.76 [0.52–1.13]	0.173	0.86 [0.59–1.25]	0.434	1.00 [0.68–1.47]	0.993
Depressiveness ±	1.85 [1.23–2.71]	**0.001**	1.50 [1.03–2.21]	**0.037**	1.57 [1.06–2.33]	**0.026**	1.65 [1.12–2.43]	**0.011**	1.78 [1.19–2.68]	**0.005**
Fatigue^†^	1.44 [0.75–2.79]	0.277	2.40 [1.24–4.65]	**0.009**	1.14 [0.57–2.30]	0.713	0.98 [0.50–1.92]	0.947	1.87 [0.83–4.24]	0.132
Constant	0.254	**0.005**	0.241	**0.004**	4.622	**0.003**	0.164	** < 0.001**	0.101	** < 0.001**
	Nagelkerke’s R^2^ = 0.163		Nagelkerke’s R^2^ = 0.151		Nagelkerke’s R^2^ = 0.136		Nagelkerke’s R^2^ = 0.106		Nagelkerke’s R^2^ = 0.106	

*d2* d2 Test of Attention, *RWT* Regensburger Verbal Fluency Test, *WMS-R* Wechsler Memory Scale-Revised, *OR* odds ratio, *CI* confidence interval

*0 = female (ref.), 1 = male

^#^Dichotomized: 0 = low/middle (levels 1–3) (ref.), 1 = high (levels 4 and 5)

+ 0 = BMI < 30 (ref.), 1 = BMI ≥ 30

^§^0 = no active smoking (former or never smoker), 1 = active smoker (occasional and current smoker); ± dichotomized based on the cutoff-value of ≥ 10: 0 =  < 10 (ref.), 1 =  ≥ 10

^†^Dichotomized based on the cutoff-value of ≥ 36: 0 =  < 36 (ref.),1 =  ≥ 36

Significant p values are marked in bold; varying sample sizes due to missing values

Higher education level was consistently associated with a decreased likelihood of cognitive impairment across all domains. Specifically, individuals with high education were less likely to show deficits in attention, working speed, lexical fluency, short-term/working memory, and in multi-domain impairment. Higher age was positively associated with the risk of impairment in attention and working speed, whereas it was inversely related to lexical fluency. Gender was significantly associated with formal lexical fluency only, with males showing a higher likelihood of impairment. Longer time since the acute SARS-CoV-2 infection was related to impairment in attention, short-term/working memory, and multi-domain impairment, but showed no significant effects on working speed or formal lexical fluency. Clinically relevant depressive symptoms were significantly associated with increased odds of cognitive impairment in all domains, with ORs ranging from 1.50 to 1.85 (*p*s < 0.05). Fatigue showed a significant association only in the domain of working speed. A BMI greater than 30 was associated with increased risk of impairment in formal lexical verbal fluency and working memory, while smoking status was not significantly related to impairment in any domain. The explained variance ranged from 10.6% (multi-domain impairment and short-term/working memory) to 16.3% (attention), as indicated by Nagelkerke’s R^2^ values. To maintain the robustness of the model, CRP was not included as a predictor due to a considerable amount of missing data.

To further examine the role of depression, we repeated the regression analyses restricted to PASC patients without clinically relevant depressive symptoms (PHQ-9 < 10; see Supplementary Table S6). In this subgroup, subclinical depressive symptoms remained significantly associated with cognitive impairment in attention and multidomain dysfunction. Higher education was significantly associated with better performance in attention, processing speed, lexical fluency, and multidomain impairment. For short-term/working memory no significant predictors were identified. Time since infection remained a significant predictor of attention and multidomain dysfunction.

## Discussion

The present study provides a comprehensive evaluation of cognitive impairment in patients with Post-Acute Sequelae of COVID-19 (PASC) and for the first time, includes a direct comparison with individuals experiencing Post-Vaccination Syndrome (PVS). The primary aims were to assess the extent of cognitive impairments in PASC, identify associated factors, and explore potential parallels with PVS. The findings support the study hypotheses and contribute valuable insights into the prevalence, patterns, and predictors of cognitive symptoms following SARS-CoV-2 infection. Preliminary results also indicate comparable cognitive patterns in PVS, warranting further investigation.

The sociodemographic characteristics of the sample, predominantly middle-aged, female, and well-educated, are consistent with those reported in other PASC studies [11, 43]. Compared to previous research [43–45], the average time since infection was longer in the present study (24.5 months), allowing for a more differentiated view of later stages of the condition. These factors should be considered when interpreting the generalizability of findings.

Nearly 80% of patients in the total sample exhibited impairments in at least one cognitive domain, with deficits most commonly observed in processing speed, attention, and lexical verbal fluency. This pattern is consistent with existing research on PASC-related cognitive dysfunction [11, 15–18]. The high rate of multidomain impairment (30.3%) is of particular concern as it may have a more profound impact on daily functioning and quality of life.

As expected, older age and lower educational attainment were consistently associated with a higher risk of cognitive impairment, supporting previous findings regarding cognitive reserve and age-related vulnerability [27, 28]. Notably, depressive symptoms emerged as a robust predictor across all cognitive domains. These findings are consistent with previous research linking depression to cognitive impairment, particularly in attention, memory, and executive function [46, 47]. It is well established that depression often co-occurs with concentration difficulties, which may contribute to broader cognitive dysfunction. In the study sample, higher levels of depressive symptoms were also associated with a greater likelihood of multidomain impairment, suggesting that depression may not only affect individual cognitive domains but also exacerbate overall cognitive burden. This underlines the need to consider depressive symptoms when evaluating cognitive complaints in PASC.

Additional analyses restricted to patients without clinically relevant depression suggest that cognitive impairment in PASC is not solely attributable to depression. Subclinical symptoms showed associations with attention and multidomain functioning, though effects were weaker than in the full sample. Higher education consistently showed protective associations with cognitive performance, particularly in attention, processing speed, and multidomain impairment, highlighting the relevance of cognitive reserve. No predictors were identified for short-term/working memory, which may indicate a stronger dependence on depressive symptomatology in this domain.

Fatigue was selectively associated with reduced processing speed, while no significant associations were observed with other cognitive domains. Similar patterns have been reported in individuals with chronic fatigue syndrome (CFS), where difficulties in sustaining attention and mental effort are commonly observed, whereas short-term memory and executive functioning often remain relatively preserved [48]. Recent findings from PASC cohorts also indicate persistent reductions in processing speed and tonic alertness that correlate with fatigue severity and remain stable over time [49]. These results suggest that fatigue in PASC may be linked to specific cognitive mechanisms, particularly affecting efficiency and sustained attention, rather than reflecting generalized impairment.

Higher BMI (> 30) was linked to deficits in verbal fluency and working memory, potentially reflecting underlying inflammatory mechanisms. This association may be explained by obesity-related inflammation and immune dysregulation, as previous studies have shown that obesity is associated with memory impairments and other neurocognitive symptoms in the context of post-COVID-19 condition [29, 50]. Moreover, chronic low-grade inflammation and impaired immune responses, which are characteristic of obesity, are thought to adversely affect brain function and systemic immunity [51]. In contrast, smoking showed no association with cognitive performance, which is consistent with some studies but not in line with others reporting inconclusive evidence of a link to cognitive symptoms in PASC [50, 52, 53]. One possible explanation could be limited variability in smoking behavior in our sample, potentially reducing statistical power.

When compared to age-adjusted normative data, participants in the study sample showed significantly reduced cognitive performance in attention, processing speed, and lexical verbal fluency. These impairments were present in both age groups (20–39 and 40–60 years), with particularly large effect sizes in the older group. In the PASC group, impairments were consistent across all domains and age groups. In contrast, significant differences in the PVS group were primarily found in the older subgroup, while younger patients showed largely comparable performance to healthy controls. Similar domain-specific impairments in PASC patients have also been reported in previous studies using healthy control comparisons [16, 54].

Elevated levels of CRP were modestly but significantly associated with impairment in attention, working memory, and multidomain dysfunction. While effect sizes were small, these associations align with emerging evidence implicating systemic inflammation in the pathophysiology of PASC-related cognitive deficits [24, 25]. In line with this, CRP has also been linked to depressive symptom severity as measured by the PHQ-9 [55], suggesting a broader role of inflammation across cognitive and affective domains. Mildly elevated CRP levels were also observed in PVS patients, although group differences were not significant. Such elevations may reflect unspecific inflammatory processes, immune dysregulation following vaccination, or comorbidities such as higher BMI. Elevated baseline CRP has been linked to vaccine responsiveness [56], suggesting that pre-existing inflammatory activity could predispose to prolonged post-vaccination symptoms. As transient CRP increases have been observed following COVID-19 vaccination as part of the acute inflammatory response [57], persistent elevations in some PVS patients cannot be excluded. While the precise mechanisms remain unclear, these findings underline the need for future studies investigating inflammatory profiles in PVS.

A significant gender difference was found in formal lexical verbal fluency, with men more frequently scoring below average scores compared to women, both in descriptive and multivariate analyses. Consistent with previous findings showing that women typically perform better on verbal tasks [58], this may indicate a gender-specific vulnerability among men in this cognitive domain within the context of PASC. No significant gender differences were observed in attention, processing speed, or working memory. Subgroup analyses confirmed that this effect was limited to the PASC group and did not apply for the PVS population. While previous studies have suggested that females may be more vulnerable to cognitive impairments in PASC due to sex-based differences in immune responses or hormonal factors [59, 60], our findings indicate a male-specific risk in lexical fluency, highlighting the need for further research into domain-specific and context-dependent gender effects.

This study also provides initial insights into cognitive functioning in individuals with PVS. Similar to the PASC group, the most frequent impairments in PVS patients were found in attention and processing speed. However, while cognitive deficits in the PASC group were consistently observed across age groups and domains, impairments in the PVS group were primarily seen in older individuals, with younger patients performing largely comparable to healthy controls. One possible explanation is that older individuals may be more vulnerable to cognitive sequelae due to age-related decline in cognitive reserve, a higher prevalence of vascular risk factors, and increased susceptibility to inflammatory processes, whereas younger patients may compensate more effectively [61, 62]. While in PASC systemic infection-related mechanisms seem to affect cognition across all age groups, cognitive impairment in PVS may represent a more gradual effect that becomes clinically evident mainly when combined with age-related vulnerabilities. Despite these age-related differences, no significant group differences emerged between PASC and PVS in depressive symptoms, fatigue severity, or CRP levels, suggesting a comparable psychological and biological symptom burden. These similarities may indicate shared underlying mechanisms contributing to cognitive dysfunction, although the current evidence base for PVS remains limited. Given the small sample size and the exploratory nature of this analysis, the results should be interpreted cautiously and warrant further investigation in larger, well-characterized PVS cohorts.

### Strengths and limitations

This study offers several notable strengths. First, all participants were recruited consecutively in a specialized Post-COVID Center by experienced clinicians, ensuring high diagnostic accuracy and supporting the representativeness of the sample within the clinical PASC and PVS population. Second, the neuropsychological test battery was both time-efficient and clinically comprehensive, targeting the core cognitive domains most commonly affected in PASC: attention, processing speed, verbal fluency, and working memory. Cognitive impairment was assessed using validated, performance-based instruments rather than self-report measures, enhancing the objectivity and clinical relevance of the findings. Third, the large sample size provides sufficient statistical power and enables subgroup analyses, including one of the first direct comparisons between PASC and PVS patients. Furthermore, the integration of psychological and biological variables, including depressive symptoms, fatigue, and CRP, allows for a multidimensional view of factors associated with cognitive functioning. The use of age-adjusted normative data for a subset of cognitive tests further strengthens the validity of group comparisons.

While this study provides valuable insights into cognitive impairment associated with PASC/PVS, several limitations should be acknowledged. The cross-sectional design limits the ability to assess the trajectory of cognitive dysfunction over time. Longitudinal research is essential to determine whether cognitive impairments persist, improve, or worsen in the long term and which factors influence cognitive impairment in a predictive manner. Although the study controlled for several psychosocial and clinical variables, other potential confounders, such as medication use or genetic predispositions, were not comprehensively evaluated. Additionally, reliance on self-reported data for variables such as BMI, depressive symptoms and fatigue introduces the possibility of reporting bias. Instead of using an age- and gender-matched control group, the study relied on normative data, which may limit the accuracy of comparisons in the neuropsychological assessments. Finally, biological markers were only available for a subset of participants, which may limit the generalizability of the findings.

Future research should prioritize longitudinal studies to monitor the course of cognitive recovery in PASC patients and to evaluate the prognostic value of biological markers such as CRP. Systematic research into PVS is also essential, as the neurocognitive symptoms reported by affected individuals may share underlying mechanisms with those observed in PASC. A better understanding of this condition is crucial for its clinical validation and for developing appropriate interventions. Additionally, the integration of multidisciplinary approaches, such as those already implemented at the Post-COVID Center in Erlangen, could further enhance patient care by addressing the medical, psychological, and cognitive aspects of recovery.

## Conclusion

In conclusion, this study provides robust evidence of significant cognitive impairments in PASC patients, with attention, working speed, and lexical fluency being the most commonly affected domains. The identification of key risk factors, including education level, age, obesity, depressive symptoms, and fatigue, offers important insights for clinicians in identifying individuals at higher risk for cognitive dysfunction. The association between elevated inflammatory markers and cognitive impairments further highlights the need for ongoing research into the biological mechanisms underlying post-viral cognitive dysfunction. Finally, the comparison with PVS offers valuable preliminary data but requires further investigation to clarify the similarities and differences between these two conditions.

## Supplementary Information

Below is the link to the electronic supplementary material.

Supplementary Material 1

## Declarations

### Avaliability of data and materials

The datasets analyzed during the current study contain sensitive patient information and are therefore not publicly avaliable due to ethical and legal restrictions. Aggregate statistical results relevant to the study are provided within the article.
