# Supplementary material for: Cognitive impairment, depression, and fatigue in post-COVID and post-vaccination syndrome: a large-scale cross-sectional study
Source: Eur Arch Psychiatry Clin Neurosci. 2025 Nov 19;276(1):221–34. doi: 10.1007/s00406-025-02144-3 (PMC12904920; doi:10.1007/s00406-025-02144-3)
Supplement: Supplementary file 1 — Supplementary Material 1 [file 406_2025_2144_MOESM1_ESM.docx]

**Table S6**

Binary logistic regression analyses in the assessed cognitive domains (respective age-adjusted scores below the test-specific norms as criterion variable) in PASC patients below the Patient Health Questionnaire-9 cut-off (<10)

| Predictors | Model 1: Attention (d2)  (n = 205) | |  | Model 2: Working speed (d2)  (n = 205) | |  | Model 3: Formal lexical fluency (RWT)  (n = 187) | |  | Model 4: Short-term/ working memory (WMS-R) (n = 215) | |  | Model 5: Multi-domain  cognitive dysfunction (≥3 tests  below average) (n = 216) | |
| --- | --- | --- | --- | --- | --- | --- | --- | --- | --- | --- | --- | --- | --- | --- |
|  | OR [CI] | p |  | OR [CI] | p |  | OR [CI] | p |  | OR [CI] | p |  | OR [CI] | p |
| Age | 1.02 [1.00-1.05] | 0.190 |  | 1.02 [1.00-1.05] | 0.088 |  | 0.97 [0.94-0.99] | **0.016** |  | 1.01 [0.98-1.04] | 0.552 |  | 1.00 [0.97-1.03] | 0.935 |
| Gender* | 0.79 [0.40-1.55] | 0.495 |  | 0.85 [0.44-1.65] | 0.631 |  | 4.25 [2.04-8.87] | **<.001** |  | 1.49 [0.76-2.91] | 0.248 |  | 1.60 [0.75-3.39] | 0.222 |
| Education# | 0.36 [0.20-0.67] | **0.001** |  | 0.34 [0.18-0.61] | **<.001** |  | 0.53 [0.28-1.00] | **0.049** |  | 0.59 [0.32-1.10] | 0.097 |  | 0.45 [0.22-0.92] | **0.028** |
| Time since acute SARS-CoV-2-infection | 1.03 [1.00-1.06] | **0.026** |  | 1.02 [1.00-1.05] | 0.115 |  | 1.00 [0.97-1.03] | 0.934 |  | 1.02 [0.99-1.05] | 0.149 |  | 1.06 [1.02-1.09] | **0.001** |
| BMI+ | 0.82 [0.38-1.80] | 0.626 |  | 0.73 [0.33-1.60] | 0.426 |  | 1.64 [0.73-3.71] | 0.232 |  | 1.12 [0.52-2.42] | 0.781 |  | 0.83 [0.33-2.04] | 0.677 |
| Smoking habits § | 1.82 [0.92-3.58] | 0.086 |  | 1.41 [0.71-2.77] | 0.324 |  | 0.94 [0.45-1.94] | 0.860 |  | 0.77 [0.37-1.59] | 0.482 |  | 0.51 [0.59-2.89] | 0.506 |
| Depressiveness ± | 1.18 [1.02-1.36] | **0.024** |  | 1.13 [0.98-1.30] | 0.097 |  | 1.06 [0.91-1.23] | 0.474 |  | 0.99 [0.86-1.15] | 0.983 |  | 1.20 [1.01-1.42] | **0.043** |
| Fatigue † | 0.81 [0.36-1.83] | 0.609 |  | 1.47 [0.66-3.31] | 0.349 |  | 1.03 [0.42-2.56] | 0.948 |  | 0.77 [0.33-1.79] | 0.535 |  | 1.11 [0.39-3.19] | 0.843 |
|  |  |  |  |  |  |  |  |  |  |  |  |  |  |  |
| Constant | 0.107 | **0.005** |  | 0.150 | **0.033** |  | 2.096 | 0.437 |  | 0.243 | 0.243 |  | 0.025 | **<.001** |
|  | Nagelkerke’s R^2^ = 0.153 | |  | Nagelkerke’s R^2^ = 0.149 | |  | Nagelkerke’s R^2^ = 0.196 | |  | Nagelkerke’s R^2^ = 0.106 | |  | Nagelkerke’s R^2^ = 0.144 | |

d2: d2 Test of Attention; RWT: Regensburger Verbal Fluency Test; WMS-R: Wechsler Memory Scale-Revised; OR = odds ratio; CI = confidence interval; * 0 = female (ref.), 1 = male; # dichotomized: 0 = low/middle (levels 1-3), 1 = high (levels 4 and 5); + 0 = BMI <30 (ref.), 1 = BMI ≥30; § 0 = no active smoking (former or never smoker), 1 = active smoker (occasional and current smoker); ± Patient Health Questionnaire-9 sum score (continuous); † dichotomized based on the cutoff-value of ≥36: 0 = <36 (ref.), 1 = ≥36; significant p values are marked in bold; varying sample sizes due to missing values.
